# Improved Monte Carlo methods for estimating confidence intervals for eleven commonly used health disparity measures

**DOI:** 10.1371/journal.pone.0219542

**Published:** 2019-07-11

**Authors:** Jaeil Ahn, Sam Harper, Mandi Yu, Eric J. Feuer, Benmei Liu

**Affiliations:** 1 Department of Biostatistics, Bioinformatics and Biomathematics, Georgetown University, Washington, DC, United States of America; 2 Department of Epidemiology, Biostatistics, and Occupational health, McGill University, Montreal, Quebec, Canada; 3 Division of Cancer Control and Population Sciences, National Cancer Institute, Bethesda, MD, United States of America; University of South Alabama Mitchell Cancer Institute, UNITED STATES

## Abstract

Health disparities are commonplace and of broad interest to policy makers, but are also challenging to measure and communicate. The Health Disparity Calculator software (HD*Calc, v1.2.4) offers Monte Carlo simulation (MCS)-based confidence interval (CI) estimation of eleven disparity measures. The MCS approach provides accurate CI estimation, except when data are scarce (e.g., rare cancers). To address sparse data challenges to CI estimation, we propose two solutions: 1) employing the gamma distribution in the MCS and 2) utilizing a zero-inflated Poisson estimate for Poisson sampling in simulation experiments. We evaluate each solution through simulation studies using female breast, female brain, lung, and cervical cancer data from the Surveillance, Epidemiology, and End Results (SEER) program. We compare the coverage probabilities (CPs) of eleven health disparity measures based on simulated datasets. The truncated normal distribution implemented in the MCS with the standard Poisson samples (the default setting of HD*Calc) leads to less-than-optimal coverage probabilities (<95%). When both the gamma distribution and the estimated mean from the zero-inflated Poisson are used for the MCS, the coverage probabilities are close to the nominal level of 95%. Simulation studies also demonstrate that collapsing age categories for better CI estimation is not a pragmatic solution.

## Introduction

Health disparities are commonplace and have received considerable attention in recent years. Better understanding the magnitude of health disparities and how they change over time has been challenging for policy makers and researchers interested in identifying effective interventions to reduce or eliminate health disparities [1, 2]. To support that goal, statistical software called HD*Calc [3] calculates eleven health disparity measures on both absolute and relative scales to measure the magnitude and trend in health disparities for specific cancer or other health outcomes. Thus far, a number of papers employed HD*Calc and have reported on the trend of disparities for a diverse range of disease outcomes [4–6]. Recently, two new disparity indices called extended relative concentration index and the absolute concentration index with directly standardized rates and their statistical inferences were developed and will be added to the next version of the HD*Calc [7].

When it comes to estimating confidence intervals (CI), HD*Calc offers two numerical approaches: the analytic approach and a Monte Carlo simulation (MCS)-based approach. The current version of the analytic Taylor-series expansion approach for relative risks, absolute risks, and Index of Disparity measures may lead to conservative confidence intervals because the dependence between the two groups being compared is neglected when deriving partial derivatives [8]. Although the MCS method performs relatively better with no such issue, its coverage probability can deviate from the nominal value (0.95) in situations with sparse data. We note that inadequate simulation procedures can result in such poor performance given that the MCS is a simulation based approach. A possible reason for this shortcoming is that a truncated normal distribution is employed in sampling age-adjusted rates (AARs) to ensure positive AARs during the Monte Carlo simulation. When the truncated normal distribution does not represent the true distribution of AARs, CI estimation will depart from the truth. In addition, Ahn et al. (2018) demonstrated that CI estimation tends to perform inaccurately when more than 25% of age-groups have cancer incidence rates of zero, often referred to as structural zeros. In this circumstance, with the standard Poisson distribution, the simulated counts are repeatedly be zero because the mean and the variance are identically zero by construction. Ahn et al. (2018) added a 1/*population*_*agegroup*_ to the Poisson mean; however, this is an *ad-hoc* modification and does not produce the true population mean. In addition, this approach often fails to address the problem of rare events. Ahn et al. (2018) also suggest collapsing age categories (e.g., 10-year rather than 5-year age groups) as a tentative solution when data are scarce, which is commonly used in categorical data analyses. However, details are lacking on: 1) how to combine age categories when different social groups have different frequencies of events and 2) the empirical performance of such a collapsing strategy.

To bridge these gaps, we propose: 1) employing a gamma distribution instead of the truncated normal distribution for the MCS-based method; and 2) using the estimated mean from a zero-inflated Poisson distribution for Poisson simulation experiments when there is an excess of zero incidence among age-groups. Through simulation studies, we illustrate how the proposed approaches perform compared to the original MCS approach. We use female breast, female brain, lung, and cervical cancer data from Surveillance, Epidemiology, and End Results (SEER) program (https://seer.cancer.gov/). We also explore the practicality of the age-category collapsing strategy by conducting exploratory simulation studies.

## Materials and methods

### 0.1 Variance estimation using the Monte Carlo simulation-based method

For the MCS approach, HD*Calc simulates age-adjusted rates (AAR), $y_{j}^{new}$, for each social group *j* using a left-truncated normal distribution, where the mean and variance are estimated using the sample mean and variance of the normal distribution. In the current version of HD*Calc, the generation of the truncated normal samples can be inefficient in that sampling from the normal distribution is repeatedly performed until a positive value is drawn. Alternatively, a gamma distribution assures a positive value and is more flexible in terms of asymmetry compared to the normal or truncated normal distribution. For very rare disease incidence or when the distribution of corresponding AARs is skewed to the left near zero, the truncated normal distribution may be less plausible. Instead the Gamma distribution can be used as an alternative for sampling AARs. In applications, the shape and scale parameters of the gamma distribution will be determined corresponding to the observed sample mean and variance of AARs. Under the Monte Carlo simulation, $y_{j}^{new}$ per each social group *j* is repeatedly simulated to obtain *B* simulated $y_{j}^{\left(b\right)},b=1,\ldots ,B$ (*B* is set at 1, 000 as a default in HD*Calc). Once each health disparity measure is obtained for all *b* = 1, …, *B*-th iterations, then the estimated health disparity measures are sorted and selected for the upper and lower bound for the CI by percentile. For example, for 95% CI the 2.5 and 97.5 percentile values are chosen.

### 0.2 Simulation studies

We aimed to assess the performance of: 1) the gamma distribution in comparison to the truncated normal distribution under the MCS approach and 2) the zero-inflated Poisson mean for the Poisson distribution in generating simulation datasets. To do so, we conducted a simulation study considering four schemes: (1) the truncated normal distribution with standard Poisson sampling (say TNP, default ub HD*Calc, where the Poisson mean is adjusted by 1/*population*_*agegroup*_); (2) the truncated normal distribution with Poisson sampling using the estimated mean from zero-inflated Poisson (TNZP); (3) the gamma distribution with standard Poisson sampling (GP); and (4) the gamma distribution with Poisson sampling using the estimated mean from zero-inflated Poisson (GZP).

For reference, we used the NCI’s SEER Program data. Without a loss of generality, we focused on female breast cancer data from Kentucky and female brain, lung, and cervical cancer from Iowa where HD*Calc has been shown to provide inaccurate CIs attributable to excessive proportions of zeros (>25%)(8). Data characteristics in terms of the number of social groups, minimum and maximum of age-adjusted rates, minimum and maximum variance of AAR, average event count per social group, proportion of zero event counts, and population size are summarized in Table 1. These four cancer types represent ordered/unordered social groups as well as a varying the number social groups used to calculate the summary measure of disparity. For example, lung cancer data comprise household income decile groups (*J* = 10) whereas female brain data comprise six unordered racial groups (*J* = 6).

**Table 1:** Five years (2008 to 2012) SEER data characteristics for female breast cancer from Kentucky and female brain, cervical, lung cancer from Iowa or Kentucky are summarized based on the number of social groups, minimum and maximum of AAR, minimum and maximum variance of AAR, average event count per social group, proportion of zero event counts among age groups across *J* social groups, population size. Each data set consists of 19 standard age groups based on 5-year intervals. For female breast and female brain cancer, six racial/ethnic groups (Hispanic (reference), White, Black, Asian, Asian Pacific Islander, and Unknown) were used. For cervical cancer and lung cancer, two ethnic groups (Spanish Hispanic Latino and Non-Spanish Hispanic Latino (reference)) and ten groups based on household deciles income in 2008 (the lowest decile group is the reference) were used, respectively.

Cancer	Female Breast	Female Brain	Cervical	Lung
States	Kentucky	Iowa	Iowa	Iowa
Number of Groups	6	6	2	10
Min AAR^†^	136.58	12.96	68.83	57.45
Max AAR^†^	1332.25	74.4	124.21	73.45
Min Var^†^	10.325	2.841	3.174	1.18
Max Var^†^	75.159	52.613	27.018	69.97
Avg. Count	165.15	5.51	14	69.97
Prop. of zeros among age-groups	0.43	0.66	0.37	0.43
Population	11,028,460	7,693,357	7,693,357	15,239,059

^†^Values are multiplied by 10^6^.

For the sampling design, we use the following terminology. Let *x*_*jk*_ denote event counts for *j*-th social group and *k*-th age group And let *y*_*j*_ be the age-adjusted rate for *j*-th social group. When *x*_*jk*_ is zero for *j*-th social group and *k*-th age group, then simulating $x_{jk}^{new}$ from *Poisson*(*x*_*jk*_) always leads to zero. In such a case, this can become a structural zero problem.

To address this situation, we propose to exploit the estimates of a zero-inflated Poisson distribution to simulate *x*_*jk*_ [9]. To be specific, the zero-inflated Poisson can be expressed as a mixture distribution, i.e., *Pr*(*x*_*jk*_ = 0) = *π*_*jk*_ + (1 − *π*_*jk*_)exp(−λ_*jk*_) and $Pr\left(x_{jk}=x\right)=\left(1-\pi_{jk}\right)\frac{\lambda_{jk}^{x}\text{exp}\left(-\lambda_{jk}\right)}{x!},x\geq 1$ where *π*_*jk*_ is the weight probability of extra zeros at *k*-th age group and social group *j* and λ_*jk*_ is the population-adjusted Poisson mean. Using SEER data from 13 states for a specific cancer allows us to observe some non-zero events at a given age group *k*. In this case, we are able to obtain estimates ${\hat{\pi }}_{jk}$ and ${\hat{\lambda }}_{jk}$ using *zeroinfl()* function in *R* package version 3.11. With estimated ${\hat{\pi }}_{jk}$ and ${\hat{\lambda }}_{jk}$, we consider a maximum of $\left(1-{\hat{\pi }}_{jk}\right){\hat{\lambda }}_{jk}$ and 1/*population*_*agegroup*_ = 1/*n*_*jk*_ as the Poisson mean in order to avoid structural zeros. That is, when *x*_*jk*_ = 0, we sample $x_{jk}^{new}$ from the $Poisson(max(\frac{1}{n_{jk}},\left(1-{\hat{\pi }}_{jk}\right){\hat{\lambda }}_{jk}))$ rather than conventional $Poisson(\frac{1}{n_{jk}})$. Resulting density plots of the proportions of zeros for four cancer types after employing this approach are illustrated in S1 Fig.

For the gamma distribution in the MCS approach, we used the scale and shape parameter corresponding to the mean and variance parameters obtained using the normal distribution. We repeated this procedures 10,000 times to generate $x_{jk}^{new}$ and obtain $y_{j}^{new}$. We used HD*Calc to calculate the eleven health disparity measures implemented in the current version (v1.2.4).

## Results

Table 2 reports the simulation results in terms of the coverage probabilities (CPs) of the eleven health disparity measures using the aforementioned four schemes. The TNP yields permissive CPs (less than 95% nominal level) in most of the eleven measures across all four cancer types, and approximately 70% to 80% CPs are observed for female brain cancer. The poor performance for female brain may seem quite surprising; however, we should note that female brain cancer data suffer from 66% zero cancer events across all age groups. There are subtle improvements in CPs with the TNZP compared to the TNP; however, the improvements are not consistently better for most health disparity measures. For example, TNZP increased the CPs for 8 out of 11 measures for female breast cancer by 0.5% to 1.6%; however, CPs for Range Difference (RD), Range Ratio (RR) and Index of Disparity (IDisp), decreased by more than 3%. The GP yields relatively better CPs than those of the TNZP and TNP by reducing the distance from 95% nominal levels across all four cancer types. For example, for lung cancer, the CPs from the GP become closer to 95% with a few exceptions (e.g., Between Group-Variance (BGV) and Theil’s Index (T)). The GZP produces CPs consistently close to 95% in most measures in the three cancer types, except for female brain cancer. Overall, the GZP appears to produce better CPs than the other three approaches, while GP outperforms TNZP. For female brain cancer data, however, all four schemes demonstrate inaccurate CI estimation.

**Table 2:** The coverage probabilities (CP) are compared with nominal coverage .95 for 11 health disparity measures under the four scenarios: The truncated normal distribution with standard Poisson sampling (TNP), the truncated normal distribution with zero-inflated mean implemented Poisson sampling (TNZP), the gamma distribution with standard Poisson sampling (GP), and the gamma distribution with zero-inflated mean implemented Poisson sampling (GZP).

	Female Breast Cancer (J = 6)				Female Brain (J = 6)			
Measures^†^	TNP	TNZP	GP	GZP	TNP	TNZP	GP	GZP
RD	0.929	0.897	0.926	0.937	0.834	0.821	0.795	0.784
BGV	0.923	0.932	0.937	0.948	0.735	0.755	0.769	0.744
ACI	0.927	0.942	0.936	0.947	0.798	0.804	0.811	0.806
SII	0.927	0.942	0.936	0.947	0.798	0.804	0.811	0.806
RR	0.904	0.859	0.927	0.939	0.723	0.776	0.796	0.783
IDisp	0.908	0.838	0.931	0.944	0.743	0.774	0.792	0.739
MLD	0.912	0.917	0.924	0.938	0.702	0.734	0.751	0.754
T	0.924	0.929	0.933	0.945	0.705	0.731	0.751	0.746
RCI	0.926	0.942	0.936	0.948	0.797	0.803	0.811	0.806
RII	0.926	0.942	0.936	0.948	0.797	0.803	0.811	0.806
KMI	0.926	0.942	0.936	0.948	0.797	0.805	0.811	0.809
	Lung Cancer (J = 10)				Cervical Cancer (J = 2)			
Measures	TNP	TNZP	GP	GZP	TNP	TNZP	GP	GZP
RD	0.841	0.973	0.948	0.951	0.929	0.931	0.943	0.940
BGV	0.903	0.896	0.912	0.913	0.931	0.932	0.944	0.950
ACI	0.940	0.941	0.947	0.949	0.930	0.932	0.944	0.947
SII	0.940	0.941	0.947	0.949	0.928	0.931	0.943	0.946
RR	0.842	0.961	0.949	0.949	0.971	0.943	0.977	0.966
IDisp	0.945	0.914	0.947	0.938	0.970	0.943	0.976	0.966
MLD	0.911	0.910	0.915	0.917	0.954	0.937	0.959	0.963
T	0.907	0.906	0.911	0.912	0.941	0.932	0.949	0.954
RCI	0.939	0.940	0.949	0.951	0.933	0.935	0.946	0.947
RII	0.939	0.940	0.949	0.951	0.933	0.935	0.946	0.947
KMI	0.939	0.940	0.949	0.951	0.936	0.928	0.941	0.953

^†^Range Difference (RD), Between Group-Variance (BGV), Absolute Concentration Index (ACI), Slope Index of Inequality (SII), Range Ratio (RR), Index of Disparity (IDisp), Mean Log Deviation (MLD), Theil’s Index (T), Relative Concentration Index (RCI), Relative Index of Inequality (RII), Kunst-Mackenbach Relative Index (KMI)

Analogous to approaches for contingency tables with zero count cells, collapsing age boundaries can serve as an alternative strategy to reduce zero incidence age groups. To evaluate whether collapsing categories can resolve the incorrect CI calculation, we conducted an exploratory study by re-analyzing previously generated simulation datasets using the TNP and GP under the MCS approach. Considering that each SEER data set consists of 19 standard 5-year window age groups, it is conceivable that younger age groups are more likely to have zero cancers. We computed the tally of cancer events in consecutive age groups for each cancer type and then we used four broad age groups (0-39, 40-54, 55-69, 70 or higher) to generate at least one cancer events in each age group. We also considered eight different age groups (0-19, 20-34, 35-50, 51-54, 55-59, 60-64, 65-74, 75 or higher) to assess sensitivity of the results to the number of collapsed age groups.

We evaluated the impact of the two strategies for collapsing age groups by comparing two summary measures, that is, averaged relative changes |*HD*_*k*′_ − *HD*_*k*_|/*HD*_*k*_, *k* = 19 (original estimates), *k*′ = 4 or 8 and its mean squared error in Table 3. We found that approximately 5% and 10% relative changes in health disparity point estimates for lung cancer and female breast cancer, respectively. As expected, collapsing to eight age groups tended to produce heath disparity measures that were closer to the original measures than those obtained from the four age group collapsing strategy in both lung and female breast cancer. For female brain and cervical cancer, however, collapsing age groups led to large departures from the original measures regardless of the number of age groups. This inconsistency suggests that collapsing age groups is unlikely to be a reliable strategy for improving CI estimates for measures of health disparity.

**Table 3:** The averaged relative changes (mean squared errors) are shown by four cancer types and the number of age-groups using the truncated normal distribution (top) and the gamma distribution (bottom) under the MCS.

	Lung Cancer		Breast (Female) Cancer		Female Brain Cancer		Cervical Cancer	
Truncated Normal distribution sampling under the MCS								
measures	4 groups	8 groups	4 groups	8 groups	4 groups	8 groups	4 groups	8 groups
RD	0.098 (0.167)	0.068 (0.163)	0.013 (0.01)	0.009 (0.01)	0.316 (0.877)	0.193 (0.636)	1.068 (382.15)	1.496 (681.037)
BGV	0.128 (0.128)	0.03 (0.093)	0.117 (0.069)	0.005 (0.049)	0.122 (0.282)	0.072 (0.208)	2.725 (303.14)	4.367 (1092.86)
ACI	0.064 (0.034)	0.004 (0.027)	0.103 (0.439)	0.101 (0.851)	0.443 (5.194)	0.739 (6.671)	0.278 (3.729)	0.499 (5.512)
SII	0.064 (0.034)	0.004 (0.027)	0.103 (0.439)	0.101 (0.851)	0.695 (20.517)	1.288 (76.939)	0.933 (391.751)	1.357 (705.161)
RR	0.015 (0.013)	0.011 (0.013)	0.326 (1.606)	0.304 (1.471)	0.409 (3.249)	0.434 (2.491)	0.007 (0.097)	0.061 (0.113)
IDisp	0.086 (0.132)	0.055 (0.125)	0.451 (2.79)	0.433 (2.501)	0.404 (4.154)	0.584 (4.495)	1.122 (423.892)	1.558 (759.088)
MLD	0.012 (0.017)	0.03 (0.007)	0.101 (0.097)	0.026 (0.081)	0.101 (0.618)	0.177 (0.602)	0.126 (0.643)	0.269 (0.918)
T	0.005 (0.011)	0.024 (0.006)	0.087 (0.07)	0.022 (0.059)	0.063 (0.271)	0.085 (0.234)	0.168 (0.92)	0.352 (1.474)
RCI	0.061 (0.033)	0.019 (0.028)	0.046 (0.163)	0.067 (0.182)	0.101 (0.677)	0.32 (1.078)	0.174 (1.075)	0.321 (1.342)
RII	0.061 (0.033)	0.019 (0.028)	0.082 (0.416)	0.107 (0.864)	0.573 (13.028)	0.93 (16.138)	0.492 (42.962)	0.775 (69.81)
KMI	0.014 (0.002)	0.002 (0.002)	0.001 (0.006)	0.003 (0.007)	0.036 (0.104)	0.073 (0.144)	7.884 (58577.526)	4.365 (1709.717)
Gamma distribution sampling under the MCS								
measures	4 groups	8 groups	4 groups	8 groups	4 groups	8 groups	4 groups	8 groups
RD	0.055 (0.041)	0.014 (0.037)	0.068 (0.131)	0.045 (0.13)	0.58 (13.53)	0.536 (9.144)	0.985 (267.024)	1.601 (1015.294)
BGV	0.06 (0.044)	0.011 (0.034)	0.071 (0.148)	0.046 (0.132)	0.523 (22.326)	0.488 (6.876)	0.742 (118.101)	1.04 (300.649)
ACI	0.056 (0.044)	0.011 (0.037)	0.07 (0.144)	0.043 (0.142)	0.441 (6.677)	0.436 (6.602)	0.714 (200.212)	1.015 (75.524)
SII	0.054 (0.04)	0.011 (0.036)	0.065 (0.152)	0.048 (0.153)	0.487 (27.836)	0.489 (19.132)	1.447 (390.293)	2.019 (663.53)
RR	0.059 (0.043)	0.013 (0.035)	0.068 (0.162)	0.05 (0.151)	0.472 (14.584)	0.558 (30.808)	0.832 (213.158)	1.647 (1062.309)
IDisp	0.056 (0.043)	0.011 (0.036)	0.07 (0.158)	0.05 (0.136)	0.425 (12.284)	0.411 (6.095)	1.641 (2025.783)	1.161 (152.202)
MLD	0.052 (0.041)	0.01 (0.035)	0.063 (0.137)	0.042 (0.126)	0.496 (9.955)	0.475 (10.131)	1.035 (322.02)	1.533 (1024.282)
T	0.06 (0.046)	0.013 (0.035)	0.063 (0.13)	0.047 (0.124)	0.358 (2.777)	0.353 (3.319)	0.605 (54.28)	0.901 (112.737)
RCI	0.056 (0.041)	0.013 (0.036)	0.06 (0.126)	0.041 (0.122)	0.454 (8.037)	0.504 (13.701)	0.698 (90.573)	1.11 (160.108)
RII	0.059 (0.044)	0.014 (0.035)	0.065 (0.128)	0.044 (0.121)	0.455 (7.248)	0.442 (5.488)	0.776 (127.05)	1.425 (362.019)
KMI	0.062 (0.045)	0.016 (0.038)	0.064 (0.127)	0.043 (0.123)	0.427 (6.13)	0.435 (7.286)	0.772 (96.267)	1.316 (346.669)

## Conclusion

This article focuses on the situation in which the Monte-Carlo simulation-based approach implemented in the HD*Calc fails to accurately estimate CIs in the context of rare cancers or cancers with excess zeroes for some age groups. We demonstrated that the gamma distribution in the MCS approach and Poisson sampling with the zero-inflated Poisson mean for simulation experiments can improve CI estimation in HD*Calc. When the gamma distribution and the zero-inflated Poisson mean estimates are employed simultaneously, we observed consistently better performance compared with HD*Calc’s current approach, which is based on the truncated normal distribution. However, when cancer events are rare (e.g., female brain cancer), the proposed solutions could not fully resolve the problem of under-coverage.

## Discussion

Although we demonstrated that utilizing the zero-inflated Poisson mean estimates for Poisson sampling alleviates inaccurate CI estimation issues to some degree, this approach may not be practical in every situation. This is because the zero-inflated Poisson approach requires data for a specific cancer from multiple populations (e.g., counties and states) in order to obtain the empirical estimate of the weight that should be placed on zero events. In addition, contrary to the gamma distribution that can be easily implemented in HD*Calc, the zero-inflated Poisson approach can be applied through simulation experiments manually. When the zero-inflated Poisson approach is not applicable, the gamma distribution, in place of the truncated normal distribution for the MCS, can be used alone. As illustrated in Table 3, reducing the number of age categories can yield huge variability in HD measures and resultant HD measures are sensitive to which age groups are combined. Both of these problems make the category-collapsing approach undesirable for better estimating CIs.

Problems with scarce events are likely to occur in the context of rare diseases and when sampling is in less-populated areas. As discussed [8], when more than 25% of age groups have zero disease incidence, HD*Calc users are advised to be cautious in making inferences from health disparity measures, including interpreting 95% confidence intervals of disparity measures. HD*Calc users may conduct simulation studies in advance to examine the validity of the confidence intervals or standard errors and then make inferences about the presence/trend of health disparities. If a sampling zero problem persists, it may helpful to reevaluate age group boundaries. And we may perform a sensitivity analysis to evaluate the effect of re-grouping age-groups and see how results varying with changes.

## Supporting information

S1 Fig Density plots of the proportions of zeros for female breast, female brain, lung, and cervical cancer data. The blue line represents the density when zero-inflated Poisson (ZIP) mean estimates is used for Poisson sampling while the red line represents the density when the standard 1/*population* is used for Poisson sampling.
(PDF)

## References

[1] Rust G and Cooper LA. How Can Practice-based Research Contribute to the Elimination of Health Disparities? J. Am. Board. Fam. Med., 2007;20:105–114. 10.3122/jabfm.2007.02.060131 17341746

[2] Strategies for Reducing Health Disparities. https://www.cdc.gov/minorityhealth/strategies2016/index.html.

[3] Breen N, Scott S, Percy-Laurry A, Lewis D, Glasgow R. Health disparities calculator: A methodologically rigorous tool for analyzing inequalities in population health. American Journal of Public Health, 2014;104:1589–1591. http://seer.cancer.gov/hdcalc 25033114 10.2105/AJPH.2014.301982 PMC4151930

[4] Rossen LM and Schoendorf KC. Measuring health disparities: trends in racial-ethnic and socioeconomic disparities in obesity among 2- to 18-year old youth in the United States, 2001-2010. Ann Epidemiol., 2012;22:698–704. 10.1016/j.annepidem.2012.07.005 22884768 PMC4669572

[5] Kent EE, Breen N, Lewis DR, de Moor JS, Smith AW, Seibel NL. US trends in survival disparities among adolescents and young adults with non-Hodgkin lymphoma. Cancer Causes Control, 2015;26:1153–1162. 10.1007/s10552-015-0609-1 26084209 PMC4556271

[6] Breen N, Lewis D, Gibson JT, Yu M, Harper S. Measuring health disparities: trends in racial-ethnic and socioeconomic disparities in obesity among 2- to 18-year old youth in the United States, 2001-2010. Cancer Causes Control, 2017;28:117–125.28083800

[7] Yu M, Liu B, Li Y, Zou J, Breen N. Statistical inferences of extended concentration indices for directly standardized rates. Stat. in Med., 2019;38:1–12. 10.1002/sim.7952 30206950

[8] Ahn J, Harper S, Yu M, Feuer EJ, Liu B, Luta G. Variance estimation and confidence intervals for eleven commonly used health disparity measures. JCO: Clin. Can. Inform., 2018. 10.1200/CCI.18.00031 PMC6873904 30652598

[9] He H, Tang W, Wang W, Crits-Christoph P. Structural zeroes and zero-inflated models. Shanghai Arch Psychiatry, 2014;25:236–242. 10.3969/j.issn.1002-0829.2014.04.008 PMC4194007 25317011

