# Supplementary material for: Improved Monte Carlo methods for estimating confidence intervals for eleven commonly used health disparity measures
Source: PLoS One. 2019 Jul 11;14(7):e0219542. doi: 10.1371/journal.pone.0219542 (PMC6622532; doi:10.1371/journal.pone.0219542)
Supplement: S1 Fig — The blue line represents the density when zero-inflated Poisson (ZIP) mean estimates is used for Poisson sampling while the red line represents the density when the standard 1/population is used for Poisson sampling. (PDF) [file pone.0219542.s001.pdf]

Supplementary : Improved Monte Carlo Methods to the estimation of the confidence intervals for eleven commonly used health disparity measures

Jaeil Ahn[1,*], Sam Harper[2], Mandi Yu[3], Eric J. Feuer[3], Benmei Liu[3]
[1] Department of Biostatistics, Bioinformatics and Biomathematics, Georgetown University, Washington, DC 20057
[2] Department of Epidemiology, Biostatistics, and Occupational health, McGill University, Montreal, Quebec H3A 1A2, Canada
[3] Division of Cancer Control and Population Sciences, National Cancer Institute, Bethesda, MD 20892
∗ *email:* ja1030@georgetown.edu

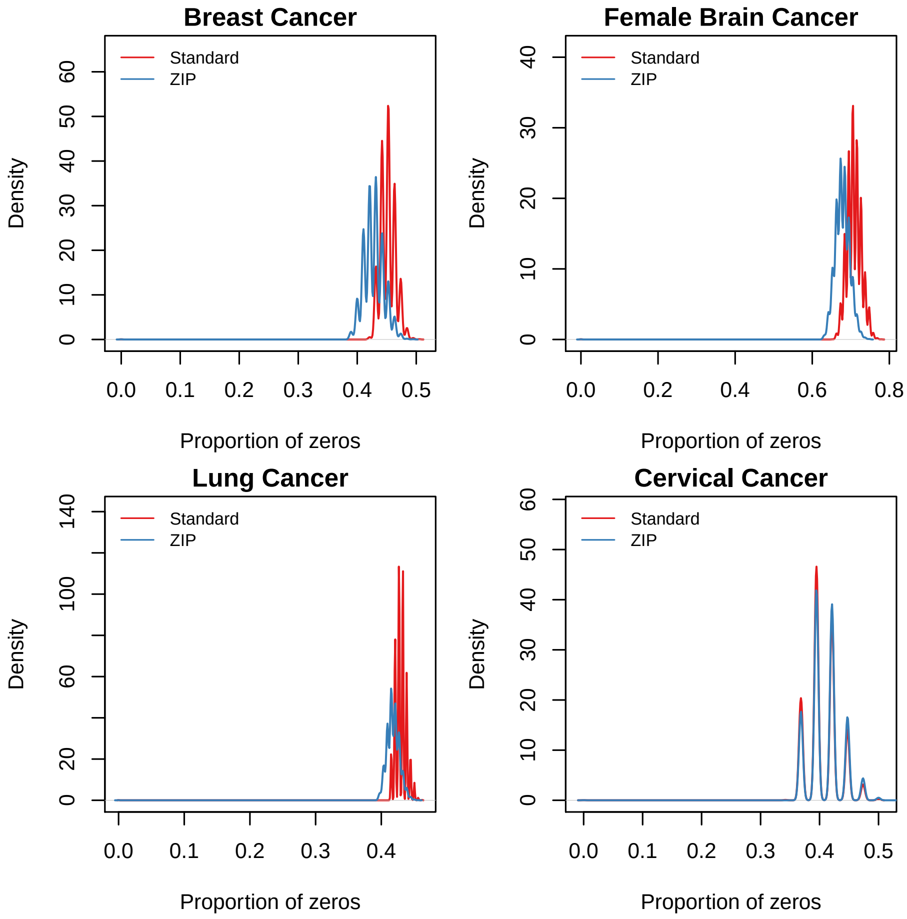

Figure 1: Density plots of the proportions of zeros for female breast, female brain, lung, and cervical cancer data. The blue line represents the density when zero-inflated Poisson (ZIP) mean estimates is used for Poisson sampling while the red line represents when the standard $1/population$ is used for Poisson sampling.
